# Exploring the Role of ROCK Inhibition in Corneal Edema Through Crosstalk Between Epithelial and Endothelial Cells

**DOI:** 10.1155/2024/9381303

**Published:** 2024-11-05

**Authors:** Lieh-Yu Yi, Hsiu-Hui Hsieh, Zhi-Qian Lin, Kai-Feng Hung, Yi-Chen Sun

**Affiliations:** ^1^School of Medicine, Tzu Chi University, Hualien, Taiwan; ^2^Department of Medical Education, Taipei Tzu Chi Hospital, Buddhist Tzu Chi Medical Foundation, New Taipei City, Taiwan; ^3^Department of Ophthalmology, Taipei Tzu Chi Hospital, Buddhist Tzu Chi Medical Foundation, New Taipei City, Taiwan; ^4^Department of Medical Research, Taipei Veterans General Hospital, Taipei, Taiwan; ^5^Department of Dentistry, School of Dentistry, National Yang Ming Chiao Tung University, Taipei, Taiwan

## Abstract

The maintenance of corneal transparency and normal vision is dependent on preservation of epithelial and endothelial cell layer homeostases. Different types of corneal injury can induce swelling and losses in transparency. Fuchs endothelial corneal dystrophy (FECD) is one type of injury that is commonly treated with rho-associated coiled–coil-containing protein kinase (ROCK) inhibitors. While their clinical benefit is apparent, certain aspects of their mechanism of action require clarification. Specifically, although topical eye drops containing ROCK inhibitors have been employed to treat corneal endothelial dysfunction–associated corneal edema, it remains unclear whether interactions between both corneal epithelial and endothelial cell contribute to mitigating clinical signs that compromise normal vision. To address this question, we first review the intricate ROCK signaling pathways and their role in modulating a variety of functions that are related to the maintenance of corneal transparency and normal vision. We also review the results of ongoing clinical trials employing current FDA-approved ROCK inhibitors, highlighting the prominent role of Y-27632 in the treatment of a variety of ocular conditions, particularly FECD, and its promising results in reversing losses in normal vision through facilitating cell proliferation and suppressing apoptosis. This review shows that the ROCK inhibitor clinical benefit is affected by their interactions between the epithelium and the endothelium. This realization makes it likely that ROCK inhibitors will be approved for use in a clinical setting to treat FECD.

## 1. Introduction

The cornea is a crucial component of the eye, serving as a protective barrier for internal structures, as well as an optic for light refraction and focusing. The structural composition of the cornea consists of five distinct layers, including the outer most epithelium, the Bowman layer, the stroma, the Descemet membrane, and the inner most endothelium. The corneal endothelium maintains corneal transparency by actively pumping excess fluid from the cornea, preventing the cornea from becoming swollen and hazy [1]. Corneal epithelial cells play a crucial role in the migration and repair of the corneal surface after injury [2]. On the other hand, the corneal endothelium is a single layer of cells located on the inner surface of the cornea. The combined function of these two layers is to maintain corneal transparency by regulating corneal deturgescence [3]. Unlike epithelial cells, corneal endothelial cells (CECs) have limited proliferative capacity in vivo, which poses a challenge for corneal regeneration and repair in cases of injury or disease.

Although the corneal epithelium and the endothelium are separated by the Bowman layer, stroma, and Descemet membrane, the coordinated function of both epithelial and endothelial cells is crucial to maintain the clarity of the cornea. Despite their importance, most current studies focus primarily on the individual corneal components, with limited research dedicated to investigate different types of interaction between these layers. This limitation is partly due to the experimental challenge of replicating the delicate corneal structure in vitro and conducting experiments involving animal models for this purpose are technically difficult. To explore the potential crosstalk between corneal epithelium and the endothelium, we adopted a different line of reasoning. We focus on the use of rho-associated coiled–coil-containing protein kinase (ROCK) inhibitor for the treatment of Fuchs endothelial corneal dystrophy (FECD), since this endeavor may show that crosstalk between corneal epithelium and endothelium contributes to its mode of action. This endeavor will provide a new perspective for improving treatment of corneal disease.

## 2. General Mechanism of ROCK-Associated Signaling

**Figure 1:**
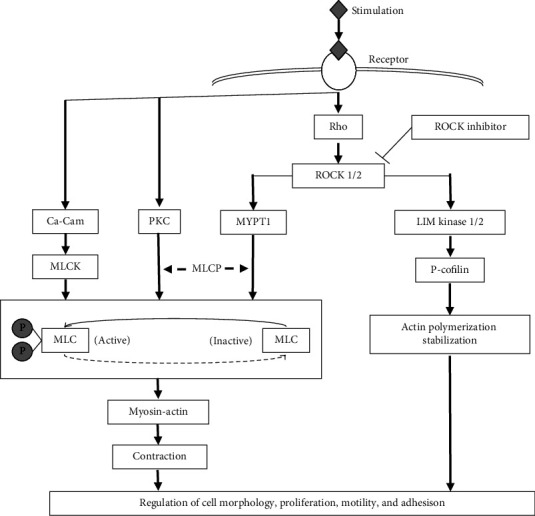
Rho kinase signaling pathway and its effect. The engagement of G protein-coupled receptors, as well as receptors for cytokines or other growth factors, initiates the activation of RhoGEF, which subsequently phosphorylates RhoA or ROCK 1/2. Predominantly, ROCK triggers two downstream pathways: firstly, the myosin light chain phosphatase (MLCP)/myosin light chain (MLC), and secondly, LIM (Lin-11, Isl-1, and Mec-3)/cofilin. Nevertheless, all these pathways harmonize in the regulation of various cellular functions including alterations in cell morphology, promotion of cell proliferation, transformation of myocytes into myofibroblasts leading to fibrosis, formation of stress fibers, and modulation of cell motility and adhesion. ROCK, Rho kinase; Ca-Cam, Ca^2+^/calmodulin; MLC, myosin light chain; MLCK, myosin light chain kinase; P-MLC, phosphorylation of MLC; PKC, protein kinase C; MLCP, myosin light chain phosphatase; MYPT1, myosin phosphatase target subunit 1; P-cofilin, phosphorylation of cofilin.

ROCK, also known as Rho kinase, is a serine–threonine protein kinase that modulates the cytoskeleton to control cell size and shape.  Figure 1 illustrates the Rho kinase signaling pathway and its effects. ROCK functions as an effector of the Ras homolog gene family member A (RhoA) protein, a Rho GTPase [4]. RhoA also regulates endothelial nitric oxide synthase (eNOS) expression and promotes basal endothelin-1 production, which have been associated with vasoconstriction and remodeling. Guanine nucleotide exchange factors (GEF), GTPase-activating proteins (GAP), and guanine nucleotide dissociation inhibitors (GDI) control this cycle between bound GDP and GTP [5]. Once activated by GTP-bound RhoA, ROCK is further modified into two isoforms: ROCK1 and ROCK2, which have approximately 65% similarity and are located on human chromosomes 18 and 2, respectively [6, 7]. Granzyme B cleaves ROCK2, whereas caspase-3 exclusively cleaves ROCK1 [8]. Subsequently, activated Rho kinase phosphorylates a variety of substrates, including myosin light chain, myosin phosphatase substrate 1, LIM (Lin-11, Isl-1, and Mec-3) kinase, protein kinase C-potentiated inhibitor protein of 17 kDa (CP1-17), calponin, and ezrin, radixin, and moesin (ERM) protein, leading to a conformational change of the trabecular meshwork tissue to promote the outflow of aqueous humor [4]. Besides, Rho kinase regulates other physiological processes, such as muscle contraction, cell migration, angiogenesis, and vasodilation. Many of these therapeutic agents, including fasudil [9], are currently in the discovery phase of clinical trials. Fasudil has demonstrated positive outcomes for patients with conditions such as systemic hypertension, vasospastic angina, stroke, and chronic heart failure [9].  Table 1 provides an overview of the clinical trials involving the application of ROCK inhibitors.

## 3. Current ROCK Inhibitor Treatment in Ophthalmology

Since ROCK is involved in cell signaling that may adversely affect many cell components of eyes, ROCK inhibitors have been of increasing importance in ophthalmology, including the treatment of glaucoma, ocular hypertension, and FECD [10].  Table 2 presents a list of ROCK inhibitors that have been approved by the US Food and Drug Administration (FDA) and the Japan Pharmaceuticals and Medical Devices Agency (PMDA) for clinical use, with Y-27632 being the most frequently prescribed. Y-27632 is acknowledged for its efficacy in the treatment of various eye conditions, including corneal diseases, FECD, retinal degenerative diseases, glaucoma, and other ocular disorders, by impeding the ROCK signaling pathway [10]. ROCK inhibitors have shown to improve the outcome of corneal epithelial and endothelial disorders [11–13]. As demonstrated in a clinical trial, Y-27632 eye drops are an effective pharmacological treatment to reduce corneal edema in patients with early-stage FECD [14]. Meanwhile, another clinical study showed that both systemic administration and topical application of ROCK inhibitors can effectively treat patients with severely damaged corneal endothelium resulting from cataract surgery by enhancing proliferation of the remaining CECs with minimal severe adverse side effects [15]. In addition to CECs, ROCK inhibitors also benefit from the treatment of corneal epithelial disease. Y-27632 has shown to effectively improve corneal epithelial wound healing by preventing inflammatory cell infiltration and promoting cell migration, proliferation, cell matrix adhesion, and cell-cell adhesion [16]. At the cell level, ROCK inhibitors have shown to induce the proliferation of remaining healthy endothelial cells [17] and the migration of CECs to promote corneal endothelial wound healing [18]. A previous study also showed that the use of Y-27632 improved CEC adhesion in vitro and increased the overall cell replication efficiency by up to 3.36 times [19]. A study by Pipparelli et al. demonstrated that human CECs cultured with ROCK inhibitors maintain their specific morphological and functional characteristics [20]. Furthermore, the research by Koizumi et al. found that Y-27632 improves the survival of frozen CECs and transplantation efficiency [21]. Mechanistically, ROCK inhibitors have also been reported to suppress corneal neovascularization and inflammation by decreasing the production of reactive oxygen species (ROS), tumor necrosis factor (TNF), vascular endothelial growth factor (VEGF), and matrix metalloproteinases 2 and 9 (MMP-2 and MMP-9) [22]. Taken together, these studies provide evidence for the various benefits of Y-27632, including promoting cell proliferation, suppressing apoptosis, maintaining cell functionality, and increasing cell survival during transplantation procedures for corneal epithelial and endothelial cells.

## 4. Corneal Epithelial and Endothelial Cells Take up Topically Applied ROCK Inhibitors

In ophthalmology, ROCK inhibitors have been administered via topical eye drops [17], intracameral injection [23], or intravitreal injection [24, 25]. With the exception of intravitreal injection, which is used primarily for retinal diseases [25], both topical application and intracameral injection of ROCK inhibitors have been reported to promote CEC regeneration [26–29]. Topical eye drops mainly penetrate the eye at the cornea and conjunctiva-sclera. The majority of drug absorption occurs at the cornea, with conjunctiva and sclera accounting for less than one fifth of all absorption [30]. Several factors, including corneal permeability, tear reflex, and drug solubility, influence uptake through the transcorneal pathway [31, 32]. Most hydrophilic drugs are capable of direct penetration through the tear film, corneal epithelial cells, and stroma to reach the corneal endothelial layer [33, 34]. However, Y-27632 has been reported to poorly penetrate through the cell membrane, limiting their paracellular transport [35]. Therefore, Y-27632 must be taken up by corneal epithelial cells through carrier-mediated facilitated diffusion in order to reach CECs [36].

## 5. Evidence Supporting the Potential Interaction Between Corneal Epithelial and Endothelial Cells

To date, although definitive evidence is not available, several interconnected signaling pathways support potential crosstalk between corneal epithelial and endothelial cells. The ephrin type-A receptor 1 (EphA1 receptor) and EphA2 receptor, members of the Eph family of receptor tyrosine kinases (RTK), are highly expressed in corneal epithelial cells. Ephrin-A1 binds with high affinity to all of the EphA receptors through the ILK-RhoA-ROCK pathway [37]. The engagement of the EphA2 receptor by Ephrin-A1 was proved to attenuate corneal epithelial cell migration [38–40]. Ephrin-A1 is abundantly expressed in CECs [41, 42], and several studies found that Ephrin-A1 can be released from cells into the extracellular environment [43–45]. As a result, CECs may possibly modulate epithelial cell migration by releasing Ephrin-A1. Similarly, a ciliary neurotrophic factor (CNTF), a member of the interleukin-6 family that can be secreted by CECs, was found to promote the activation and proliferation of corneal epithelial stem/progenitor cells, thus facilitating wound healing of the corneal epithelium [46]. Aside from endothelial cells influencing epithelial cells, there is a reciprocal influence where epithelial cells can also impact on endothelial cell function. Hatou et al. found that human embryonic stem cells can be differentiated into CECs in coculture with human corneal stroma cells and lens epithelial cell-conditioned medium (LECCM), implying that corneal stromal cells and lens epithelial cells may release soluble factors capable of facilitating corneal endothelial differentiation [47, 48]. Extending this potential interaction between epithelial cells and endothelial cells to other components of the eyes, retinal pigmented epithelial cells and choroidal endothelial cells were also shown to have a crosstalk via the annexin A1 (ANXA1)/formyl peptide receptor 2 (FPR2)/Src homology region 2-containing protein tyrosine phosphatase 2 (SHP2)/nucleotide-binding domain, leucine-rich–containing family, pyrin domain–containing-3 (NLRP3) signaling to promote choroidal neovascularization [49]. Importantly, a recent study utilizing single-cell transcriptomics to construct cell-cell communication networks of the mouse cornea found that corneal epithelial cells, serving as the most prominent source and signaling targets of epidermal growth factor (EGF) ligand, predominantly mediated EGF-related intercellular communications. Furthermore, this study also revealed that CECs serve primarily as a source of TGF-*β*, exerting both autocrine and paracrine effects on epithelial cells expressing TGF-*β*1 receptors [50]. Collectively, these studies suggest that corneal epithelial and endothelial cells probably actively interact to maintain corneal physiological and pathological homeostasis processes.

## 6. Evidence Supporting the Potential Epithelial–Endothelial Cell Interaction in Other Organs and Systems

While still in its infancy of research, several studies imply the existence of crosstalk between epithelial and endothelial cells in different tissues under various physiological or pathological conditions [51, 52]. As exemplified by a study on severe acute respiratory syndrome coronavirus 2 (SARS-CoV-2) infection, virus invasion into alveolar epithelial cells and subsequent release of cytokines can modulate the response of nearby endothelial cells, which documents interaction between these two cell types within the respiratory system [52]. Another study used double-stranded RNA to mimic viral infection, showing that simulated infection in mucosal epithelial cells promotes the release of TNF-a, which subsequently stimulates the production of chemokine (C-X3-C motif) ligand 1 (CX3CL1) and the expression of adhesion molecules in mucosal endothelial cells and highlights the interactions between epithelial and endothelial cells in airway mucosa during infections [51]. In addition, a previous study using a bilayer model and advanced microscopy techniques showed that endothelial cells in the airway mucosa induce a robust immune response when the mucosal epithelium encountered air particulates [53]. Furthermore, a recent study also showed that the crosstalk between tubular epithelial cells (TECs) and glomerular endothelial cells (GECs), which are closely located within the renal system, plays a crucial role in the progression of diabetic kidney disease (DKD) [54]. Therefore, the crosstalk between epithelial cells and endothelial cells is an essential response of physiological or pathological cellular responses.

## 7. Conclusion

As the cornea comprises various cell types that collectively maintain homeostasis to preserve its transparency, it is possible that communication exists between these cells, particularly corneal epithelial and endothelial cells. However, this concept is not definitively proven and only inferred from studies. Understanding the crosstalk between corneal epithelial and endothelial cells is not just an academic exercise; it has profound implications for improving corneal health, controlling disease progression, and developing novel treatment strategies. By providing a comprehensive overview of this topic, we hope to stress its significance in abetting ophthalmological research.

## Figures and Tables

**Table 1:** Clinical trials of ROCK inhibitors.

Rock inhibitors	NCT number	Condition or disease	Intervention	Current phase
Y-27632	NCT05309135	Corneal edema	Drug: HCEC-1	Phase 1
CPL409116	NCT04670757	Healthy	Drug: oral CPL409116	Phase 1
Fasudil	NCT04734379	• Progressive supranuclear palsy • Corticobasal syndrome	Drug: oral fasudil	Phase 2
Fasudil	NCT04793659	Dementia	Drug: oral fasudil	Phase 2
Fasudil	NCT03753269	ST segment elevation myocardial infarction	Drug: fasudil hydrochloride	Phase 4
Fasudil	NCT00498615	Raynaud Scleroderma	Drug: oral tablets of fasudil	Phase 3
Fasudil	NCT03792490	Amyotrophic lateral sclerosis	Drug: fasudil IV solution	Phase 2
Fasudil	NCT04191954	Retinopathy of prematurity	Drug: fasudil eye drops	Phase 2
Fasudil	NCT03391219	Retinal vein occlusion	Drug: injection combined bevacizumab and fasudil	Phase 3
Fasudil	NCT01823081	Diabetic macular edema	Drug: intravitreal injection of fasudil and bevacizumab	Phase 3
Fasudil	NCT00120718	• Atherosclerosis • Hypercholesterolemia	Drug: fasudil	Phase 2
Fasudil	NCT03404843	Cardiovascular diseases	Drug: fasudil hydrochloride	Phase 2
Ripasudil	NCT04621136	Retinopathy of prematurity	Drug: ripasudil ophthalmic solution 0.4%	Phase 1 Phase 2
Ripasudil	NCT03813056	Fuchs endothelial dystrophy	Drug: glanatec Drug: optive, ophthalmic solution Procedure: descemet membrane endothelial keratoplasty	Phase 2
Ripasudil	NCT05528172	Corneal edema after cataract surgery	Drug: ripasudil	Phase 3
PHP-201	NCT04863365	• Primary open angle glaucoma • Ocular hypertension	Drug: placebo ophthalmic solution Drug: PHP-201 ophthalmic solution	Phase 2
Belumosudil	NCT04735822	Healthy	Drug: oral suspensions of belumosudil Drug: oral tablets of belumosudil	Early phase 1
INS-115644	NCT00443924	• Ocular hypertension • Open angle glaucoma	Drug: INS115644 ophthalmic solution	Phase 1
LX7101	NCT01528111	• Primary open-angle glaucoma • Ocular hypertension	Drug: LX7101	Phase 1 Phase 2

*Note:* To learn more about the studies above, refer to them using the ClinicalTrials.gov identifier (NCT number).

**Table 2:** Commonly known rho-kinase inhibitors and their usage.

ROCK inhibitors	Clinical use	Main route of administration	Mechanism and pharmacodynamics	Side effects	Note
Y-27632	Treatment of FECD	Topical (ophthalmic solution)	Corneal clearing and visual improvement	Symptomatic hypotension	• The first small molecule ROCK inhibitor • Approved for clinical use in the United States
Ripasudil (K-115)	Treatment of glaucoma and ocular hypertension	Topical (ophthalmic solution)	Reduce IOP	Mild conjunctival hyperemia	• A derivative of fasudil • The first ROCK inhibitor approved for clinical use • Approved for clinical use in Japan and the United States
Netarsudil (AR-13503)	Treatment of glaucoma and ocular hypertension, specifically in the United States	Topical (ophthalmic solution)	Reduce IOP	Conjunctival hyperemia and corneal verticillata, instillation site pain, and conjunctival hemorrhages	• Both an RKI and a norepinephrine transport inhibitor • Approved for clinical use in Japan and the United States
Fasudil	Treatment of vasospasms following subarachnoid hemorrhage, specifically in Japan	Intravenous infusion	Suppress cerebral vasospasm and associated cerebral ischemic symptoms	No severe side effects	Approved for clinical use in Japan and China
Belumosudil	Treatment of chronic graft versus host disease	Oral	Inhibit pro- and anti-inflammatory immune cell responses	Infections	Approved for clinical use in the United States

## Data Availability

The references used to support the findings of this study are included within the article.
